# REPRESENT: REPresentativeness of RESearch data obtained through the ‘General Informed ConsENT’

**DOI:** 10.1186/s12910-022-00877-7

**Published:** 2023-02-13

**Authors:** Cristina Bosmani, Sonia Carboni, Caroline Samer, Christian Lovis, Thomas Perneger, Angela Huttner, Bernard Hirschel

**Affiliations:** 1 Faculty of Medicine, Clinical Research Center, Geneva University Hospitals, Rue Gabrielle-Perret-Gentil 4, 1205 Geneva, Switzerland; 2 Clinical Pharmacology and Toxicology Department, Geneva University Hospitals, Geneva, Switzerland; 3 Faculty of Medicine, Division of Infectious Diseases, Geneva University Hospitals, Geneva, Switzerland; 4 Division of Medical Information Sciences, Geneva University Hospitals, Geneva, Switzerland; 5 Division of Clinical Epidemiology, Geneva University Hospitals, Geneva, Switzerland; 6 Health Department of the Canton of Geneva, Geneva Cantonal Ethics Commission, Geneva, Switzerland

**Keywords:** Informed consent, Consent bias, Volunteer bias, External validity, Representativeness

## Abstract

**Background:**

We assessed potential consent bias in a cohort of > 40,000 adult patients asked by mail after hospitalization to consent to the use of past, present and future clinical and biological data in an ongoing ‘general consent’ program at a large tertiary hospital in Switzerland.

**Methods:**

In this retrospective cohort study, all adult patients hospitalized between April 2019 and March 2020 were invited to participate to the general consent program. Demographic and clinical characteristics were extracted from patients’ electronic health records (EHR). Data of those who provided written consent (signatories) and non-responders were compared and analyzed with R studio.

**Results:**

Of 44,819 patients approached, 10,299 (23%) signed the form. Signatories were older (median age 54 [IQR 38–72] vs. 44 years [IQR 32–60], *p* < .0001), more comorbid (2614/10,299 [25.4%] vs. 4912/28,676 [17.1%] with Charlson comorbidity index ≤ 4, *p* < .0001), and more often of Swiss nationality (6592/10,299 [64%] vs. 13,813/28,676 [48.2%], *p* < .0001).

**Conclusions:**

Our results suggest that actively seeking consent creates a bias and compromises the external validity of data obtained via ‘general consent’ programs. Other options, such as opt-out consent procedures, should be further assessed.

**Supplementary Information:**

The online version contains supplementary material available at 10.1186/s12910-022-00877-7.

## Background

Good clinical research practice—and the laws of many nations—require that patients grant informed consent in writing before their clinical data or biological material be used for research purposes. The informed consent form (ICF) documents the patient’s free will regarding the use of his/her own personal data; it is thus not only a legal but also an ethical requirement for most medical research. In this context, and in an era of increasingly ‘big data’, Swiss tertiary-care hospitals have collaborated to obtain written consent prospectively from both in- and outpatients. Through the ‘General Consent’ program, patients are approached to consent to the use of their past, present and future data for purely observational analysis, either in person or by mail. Unfortunately, most patients do not respond. Patients granting consent are a minority; their demographic and clinical characteristics may thus differ significantly from those of non-responders.

It has been well documented on a small scale that randomized clinical trials (RCTs) exclude patients who are sicker, more complicated, and whose follow-up is limited [1, 2]. The external validity of observational studies is also questionable. Certain demographic and/or clinical factors may influence the patient’s decision to grant proactive, opt-in consent, thus creating a well-documented ‘consent bias’ [3–7]. Age, sex and socioeconomic status are factors that have been associated with patients’ decision to grant consent. Disease severity has also been shown to influence willingness and/or ability to provide consent [4–7].

Understanding the representativeness of patients included in clinical research is important for the medical community, whose members increasingly rely on general-consent programs like that of the Swiss for ongoing research and policy decisions. We compared socioeconomic, demographic and clinical characteristics of those who consented versus those who did not in a cohort of > 40,000 patients of the Geneva University Hospital invited to provide general consent for the use of their clinical data in research.

## Methods

### Study design, setting, and participants

This single-center retrospective cohort study included all adult patients (≥ 18 years) who were hospitalized at the University Hospital of Geneva (HUG) between April 2019 and March 2020, thereafter invited by mail to participate in the hospital’s ‘general consent’ program (Additional file 1), and either provided consent (signatories) or did not respond (non-responders). Patients who actively refused the general consent were excluded, in order to comply with our hospital’s current policy on data protection. In addition, patients whose invitations to participate were returned (recipient not found) were also excluded, as they never received the information and thus were unable to provide any consent. Demographic and clinical data were extracted from patients’ electronic health records (EHR).

The HUG is a Swiss university medical network of eight hospitals, some 2000 beds, > 55,000 annual admissions, and > 1 million treated outpatients per year. The largest tertiary care center in Switzerland, the HUG serves a region with > 500,000 international and very mobile inhabitants. The ‘general consent’ program was launched at the HUG in 2017. Initially, individual wards and clinics were tasked with approaching patients for general consent during hospitalizations or ambulatory encounters. Due to uneven levels of engagement, in 2020 the general consent program switched mainly to regular home mailings of the general consent form to all patients who had been recently hospitalized (any hospitalization from April 2019 onwards). The general consent form is available in French, German, Italian, English, Spanish, Portuguese, Russian, Arabic, Albanian, Romanian and Georgian.

### Primary and secondary outcomes

The primary outcome was the difference between signatories and non-responders in three demographic characteristics: age (years), sex (percentage of male versus female), and country of origin (percentage of Swiss versus other). Additional outcomes included differences in language spoken; country of origin; marital status; religion; number of hospitalizations; lengths of stay (LOS); and comorbidity levels (by Charlson comorbidity and Elixhauser comorbidity indices).

### Statistical analysis

There was no sample size calculation; all adult patients invited by mail to participate in the ‘general consent’ program (without postal return of the invitation) were included. Continuous data are presented as the median with interquartile range and categorical data as frequency counts and percentages. Mortality through October 2021, the most recent time point for which data were available, was assessed. Missing data are reported throughout. Comparisons between groups were performed with the Student’s *t*-test for continuous data and Χ^2^ or Fisher’s exact test for categorical data. Associations with p values < 0.05 were considered significant (two-sided). Univariable and multivariable logistic regression models were used to determine associations, if any, between demographic or clinical baseline factors and general-consent response. For multivariable logistic regression, all variables that were significant (p value < 0.05) in univariable analyses were added to the model. Only variables that were significant were kept in the final model. All data were analyzed using R language and R studio (4.1.0, www.R-project.org/), including the packages: ‘ggplot2’, ‘ggpubr’ and ‘comorbidity’.

## Results

### Response and consent rates

**Fig. 1:**
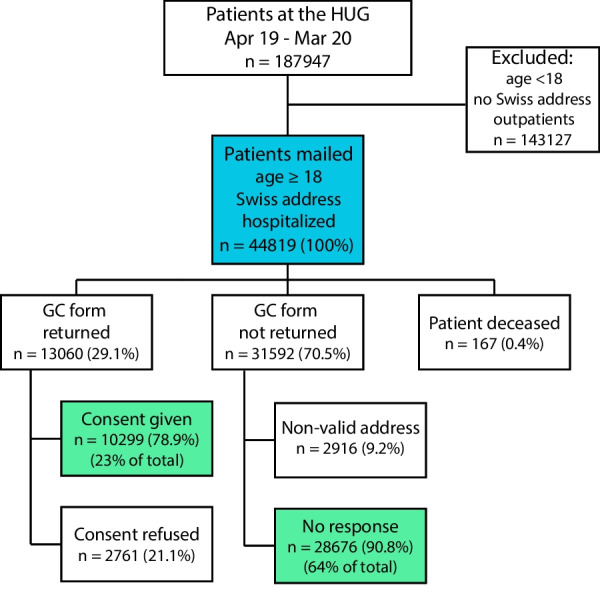
Flowchart of REPRESENT. About 45,000 patients were mailed the GC (general consent) form (blue). After excluding refusals and non-delivered forms (patient deceased or non-valid address), a total of 38,975 patients were included (light green)

Some 190,000 patients had one (or more) medical encounter(s) at the HUG between April 2019 and March 2020. Of these, 44,819 patients (roughly 24%) were hospitalized, had a Swiss residential address and thus received the general consent form by mail (Fig. 1). The overall response rate from these patients was 13,060/44,819 (29.1%). The majority of responses were positive: 10,299/13,060 (78.9%) respondents granted consent (‘signatories’), while 2,761/13,060 (21.1%) actively refused. In total, 28,676/44,819 (64.0%) were non-responders, after exclusion of patients that refused or never received the consent form (because of death or invalid address).

### Demographic characteristics of signatories and non-responders

While signatories were older (median age 54 (IQR 38–72) vs. 46 years (IQR 33–63), *p* < 0.0001), the overall percentage of women in either group did not differ significantly (5,763/10,299 [56.0%] vs. 16,144/28,676 [56.3%], *p* = 0.56; Table 1). Among younger patients (18–40 years), however, signatories were more likely to be women (1,894/2817 [67.2%] vs. 7,021/12,002 [58.5%], *p* < 0.0001), whereas the opposite was observed among older patients (2,054/4,209 [48.8%] vs. 4,076/7,311 [55.8%], *p* < 0.0001). Signatories were more often of Swiss nationality (6,592/10,299 [64.0%] vs 13,813/28,676 [48.2%], *p* < 0.0001) and French-speaking (7,915/10,299 [76.9%] vs 19,285/28,676 [67.3%], *p* < 0.0001). Married patients were more likely to consent (4,915/10,299 [47.7%] vs. 11,621/28,676 [40.5%], *p* < 0.0001), as were patients not practicing any religion (1,455/10,299 [14.1%] vs. 3,438/28,676 [12%], *p* < 0.0001). Finally, patients living in higher-income townships of the canton of Geneva were more likely to grant consent (643/9,360 [6.9%] vs 1,391/26,562 [5.2%], *p* < 0.0001).

**Table 1:** Baseline characteristics of patients approached for general consent

Characteristic	Overall n = 38,975	Signatories n = 10,299	Χ^2^/*t*-test *p* Value
Sex			
Women, *n* (%)	21,907 (56.2)	26.3	0.56
Men, *n* (%)	17,068 (43.8)	26.6	
Median age, years (IQR)	46 (33–63)	54 (38–72)	< 0.0001
Age groups and sex			
18–40 y			
Women, *n* (%)	8915 (60.2)	21.2	< 0.0001
Men, *n* (%)	5904 (39.8)	15.6	
60 + yo			
Women, *n* (%)	6130 (53.2)	33.5	< 0.0001
Men, *n* (%)	5390 (46.8)	40.0	
Country of origin			
Switzerland, *n* (%)	20,405 (52.4)	32.3	Swiss *vs.* other: < 0.0001
Other, *n* (%)	18,454 (47.3)	19.9	
Spain, *n* (%)	1348 (3.5)	22.1	
France, *n* (%)	2174 (5.6)	26.1	
Italy, *n* (%)	1879 (4.8)	26.9	
Portugal, *n* (%)	3211 (8.2)	20.3	
N/A, *n*	116		
Religion			
Religion, *n* (%)	19,144(49.1)	26.8	Religion *vs.* no religion: < 0.0001
No religion, *n* (%)	4893 (12.6)	29.7	
Refuse to respond, *n* (%)	1199 (3.1)	28.8	
N/A, *n*	13,739		
Marital status			
Married, *n* (%)	16,536 (42.4)	29.7	Married *vs.* single: < 0.0001
Single, *n* (%)	14,461 (37.1)	21.2	
Widowed, *n* (%)	2051 (5.3)	30.6	
Other, *n*	5760	28.6	
N/A, *n*	167		
Median household income of the commune of residence			
0-100 k CHF, *n* (%)	5283 (14.7)	23.5	> 200 k *vs.* 0-100 k: < 0.0001
100-200 k CHF, *n* (%)	28,605 (79.6)	26.1	
> 200 k CHF, *n* (%)	2034 (5.7)	31.6	
Spoken language			
French, *n* (%)	27,200 (69.8)	29.1	French *vs.* other: < 0.0001
Other, translated, *n* (%)	9227 (23.7)	20.9	
Other, not translated *n* (%)	2120 (5.4)	15.4	
N/A, *n*	429		

### Clinical characteristics of signatories and non-responders

The number of patients who died after the general consent form was sent was similar among signatories and non-responders (203/10,299 [2.0%] vs. 550/28,676 [1.9%], *p* = 0.77; Table 2). Signatories had significantly longer LOS (12.7 [IQR 0.8–47.0] vs. 8.8 days [IQR 0.3–40.6], *p* < 0.0001). Signatories had more comorbidities: 2,614/10,299 patients (25.4%) vs. 4,912/28,676 (17.1%; *p* < 0.0001), had a Charlson comorbidity index score between 1 and 4, and 3,064 of 10,299 patients (29.8%] vs. 6,457 of 28,676 (22.5%), (*p* < 0.0001) had an Elixhauser comorbidity index score between 1 and 4.

**Table 2:** Clinical characteristics

Characteristic	Overall	Signatories % or median (IQR)	Χ^2^/*t*-Test p value
Patient status			
Alive, *n* (%)	38,222 (98.1)	26.4	0.77
Deceased, *n* (%)	753 (1.9)	27.0	
Number of hospital encounters per patient			
1–5, *n* (%) 6–10, *n* (%) > 10, *n* (%)	20,945 (53.7) 10,429 (26.8) 7601 (19.5)	24.7 27.7 29.5	1–5 *vs* > 10: < 0.0001
Length of stay (cumulated days per patient), median (IQR)	50.3 (1.2–304.0)	72.7 (2.8 – 351.0)	< 0.0001
Charlson comorbidity index			
Score 0, *n* (%)	30,969 (79.5)	24.2	Score 0 *vs* 1 – 4: < 0.0001
Score 1–4, *n* (%)	7526 (19.3)	34.7	
Score ≥ 5, *n* (%)	480 (1.2)	36.7	
Elixhauser comorbidity index			
Score 0, *n* (%)	26,356 (67.6)	23.6	Score 0 *vs* 1 – 4 < 0.0001
Score 1–4, *n* (%)	9521 (24.4)	32.2	
Score ≥ 5, *n* (%)	3098 (7.9)	33.2	

### Consent rates by pathology

**Fig. 2:**
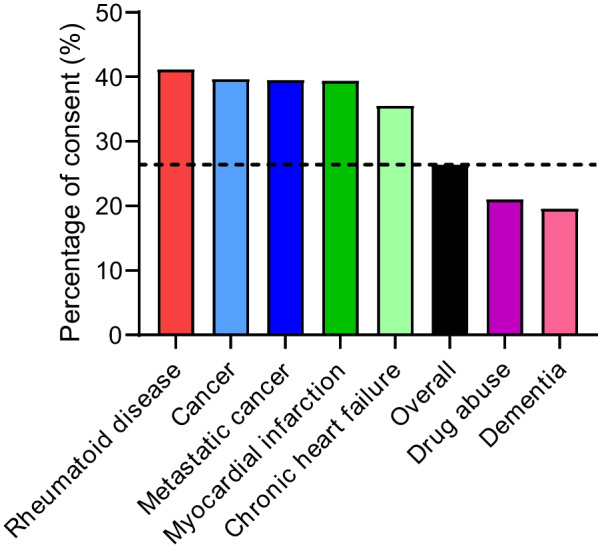
Percentage of consent among patients with selected diagnoses. Shown in black and as a dashed line the overall percent of consent of the whole dataset

Signatories had cancer more frequently than non-responders (785/10,299 [7.6%] vs. 1193/28,676 [4.2%, *p* < 0.0001]), as well as chronic heart failure (592/10,299 [5.7%] vs. 1076/28,676 [3.8%], *p* < 0.0001), myocardial infarction (389/10,299 [3.8%] vs. 598/28,676 [2.1%], *p* < 0.0001), and rheumatoid disease (129/10,299 [1.3%] vs. 184/28,676 [0.6%], *p* < 0.0001). In contrast, only 153/10,299 (1.5%) and 143/10,299 (1.4%) signatories suffered from drug abuse and/or dementia vs. 577/28,676 (2.0%, *p* < 0.0001) and 587/28,676 (2.0%, *p* < 0.0001) non-responders, respectively. Details are shown in Table 3 and Fig. 2.

**Table 3:** Distribution of selected diagnoses. 0, patients without the selected disease; 1, patients with the disease; OR, odds ratio

Characteristic	Overall	Signatories (%)	Χ^2^ *p* Value
Rheumatoid disease			< .0001
0, *n* (%)	38,662 (99.2)	26.3	
1, *n* (%)	313 (0.8)	41.2	
Cancer			< .0001
0, *n* (%)	36,997 (94.9)	25.7	
1, *n* (%)	1978 (5.1)	39.7	
Metastatic cancer			< .0001
0, *n* (%)	38,401 (98.5)	26.2	
1, *n* (%)	574 (1.5)	39.5	
Myocardial infarction			< .0001
0, *n* (%)	37,988 (97.5)	26.1	
1, *n* (%)	987 (2.5)	39.4	
Chronic heart failure			p < .0001
0, *n* (%)	37,307 (95.7)	26.0	
1, *n* (%)	1668 (4.3)	35.5	
Drug abuse			p < .0001
0, *n* (%)	38,245 (98.1)	26.5	
1, *n* (%)	730 (1.9)	21.3	
Dementia			p < .0001
0, *n* (%)	38,245 (98.1)	26.6	
1, *n* (%)	730 (1.9)	19.6

### Modeling consent

In a multivariable analysis including all characteristics described above, the strongest independent predictors (*p* < 0.0001) of granting consent were increasing age, Swiss nationality, French as first language, not having any particular religion, being married, a higher comorbidity score, and a higher number of hospital encounters (Table 4).

**Table 4:** Independent predictors of granting consent

	Odds ratio	95% CI	*p*-Value
18–40 y, female	0.75	0.69–0.81	< 0.0001
18–40 y, male	0.55	0.50–0.61	< 0.0001
60 + y, female	1.25	1.14–1.36	< 0.0001
60 + y, male	1.57	1.44–1.71	< 0.0001
Nationality—not Swiss	0.63	0.60–0.67	< 0.0001
Practicing a religion	0.80	0.75–0.86	< 0.0001
Unknown religion	0.85	0.79–0.92	< 0.0001
Marital status—single	0.81	0.76–0.86	< 0.0001
Marital status—widowed	0.73	0.66–0.82	< 0.0001
Language—not French	0.76	0.71–0.81	< 0.0001
Number of hospital encounters—1—5	0.83	0.77–0.88	< 0.0001
Charlson comorbidity index score—0	0.79	0.63–0.99	0.04
Elixhauser comorbidity index score—0	1.25	1.10–1.41	< 0.0001
Elixhauser comorbidity index score—1–4	1.31	1.18–1.45	< 0.0001

## Discussion

The purpose of a general-consent program is to obtain and document patients’ consent to the use of their clinical and biological data in the larger effort to strengthen the evidence base and thus continually improve medical care. Yet our results demonstrate important, unintended consequences: actively seeking opt-in consent from patients—an essential step in guaranteeing their rights and self-determination—may create a bias itself, thus potentially compromising the external validity of the data obtained.

In this cohort of > 40,000 patients admitted to hospital in the course of one year, we detected important differences in clinical and demographic characteristics of those who grant consent—and thus, whose data are being used for myriad ongoing and future research projects—and those who do not: a silent but massive majority (64%). Consenters are significantly older and enjoy a higher socioeconomic status. They are more likely to hold Swiss nationality, speak the dominant language, and profess no particular religion. Clinically, consenters are more comorbid, with longer recent lengths of hospital stay, probably correlating with their older age. They are significantly more likely to suffer from cancer and chronic heart disease, but less likely to suffer from drug abuse or dementia.

This is the largest cohort analyzed to date. In a systematic review assessing characteristics of patients approached for access to medical records for 17 different prospective observational studies [3], the majority (66%) of patients granted consent (in stark contrast to our cohort, with only 23%). Those granting consent to access their medical records differed significantly from non-participants across all 17 different studies, although in an inconsistent manner [3]. A smaller cohort from Ireland published two years later found a selection bias similar to what we observe after seeking consent for access to medical records: consenters were older and socioeconomically more affluent [4]. Other small studies have also shown that consenters differ from non-consenters, especially when considering their health status [5–7]. Due to the recent nature of our data, we are unable to show differences in survival times after granting consent. Data from Canadian stroke and New Zealander breast cancer registries demonstrate that patients granting consent have lower mortality rates and less advanced disease [5, 7]. It should be noted that there are differences in the target population of each one of these studies, carried out in different countries, as well as differences in the way consent was sought (by mail or in person, with or without follow-up, etc.). Nonetheless, these reports support the hypothesis that opt-in consent approaches introduce a bias in observational studies, although the extent and nature of these biases differ from study to study. Overall, our research suggests that, although they constitute a large percentage of the Geneva population, younger, more foreign and more underprivileged patients may be underrepresented in studies derived from the General Consent program.

These findings support exploration of other models to ensure patients’ rights—to both privacy and to health care informed by clinical research that is unbiased and otherwise methodologically sound. One model may be a multilateral strategy including increased ethics and legal oversight (to ensure consistent anonymization of data, among other aspects), regular provision of study information to all patients, and an opt-out process that does not require active signatures and returning of forms [8]. For now, the requirement for written informed consent clearly excludes the clinical experiences of the majority of patients. Prioritizing patient privacy is critical, but it must be balanced with patients’ other rights, including that of access to well-informed health care.

Our study has limitations. It is retrospective. By definition, only patients surviving their hospitalization could be addressed. Importantly, we were unable to test or choose the method of obtaining informed consent, here based on a mailing approach. This method could account for the low participation rate compared to other studies [3], as patients may forget or misplace the letter, or be unable to understand the language of the consent (initially sent in French), thus introducing a bias, as, for instance, non-responders are largely non-Swiss nationals. The mailing approach has only recently replaced the in-person approach initially used in the hospital, which was highly department- and person-dependent. Currently, Swiss hospitals are developing together a generalized electronic method to collect consent in the near future. Finally, because there was no follow-up after the mailing, it is not possible to know the reasons for no-response, this could add a further bias to our study.

More longitudinal analyses will need to be performed to assess whether signatories with comorbidities are in overall ‘better health’ than non-responders. Further, we are not currently able to access data from active refusers (those who return the consent form only to refuse access to their data). This is a population of patients that is understudied and obviously underrepresented in current research.

## Conclusions

Patients granting consent to the use of their clinical data for observational study are older, enjoy a higher socioeconomic status, and more likely to suffer from cancer and chronic heart disease; those with dementia and drug abuse are underrepresented. In order to increase external validity and clinical relevance of current datasets and thus decrease the probability of misleading or false research results, alternative strategies should be explored. Public-health policymakers may consider strategies such as collective, anonymized datasets obtained through informed, opt-out consent procedures tightly controlled with institutionally mandated ethics oversight, in order to guarantee anonymity and confidentiality.

## Supplementary Information

**Additional file 1**. General Consent brochure in English.

## Data Availability

The datasets used and/or analyzed during the current study are available from the corresponding author on reasonable request.
