# Supplementary material for: REPRESENT: REPresentativeness of RESearch data obtained through the ‘General Informed ConsENT’
Source: BMC Med Ethics. 2023 Feb 13;24:10. doi: 10.1186/s12910-022-00877-7 (PMC9926654; doi:10.1186/s12910-022-00877-7)
Supplement: Supplementary file 1 — Additional file 1. General Consent brochure in English. [file 12910_2022_877_MOESM1_ESM.pdf]

Anglais

ASSISTING RESEARCH

Consent for the re-use of health-related data and samples for research purposes

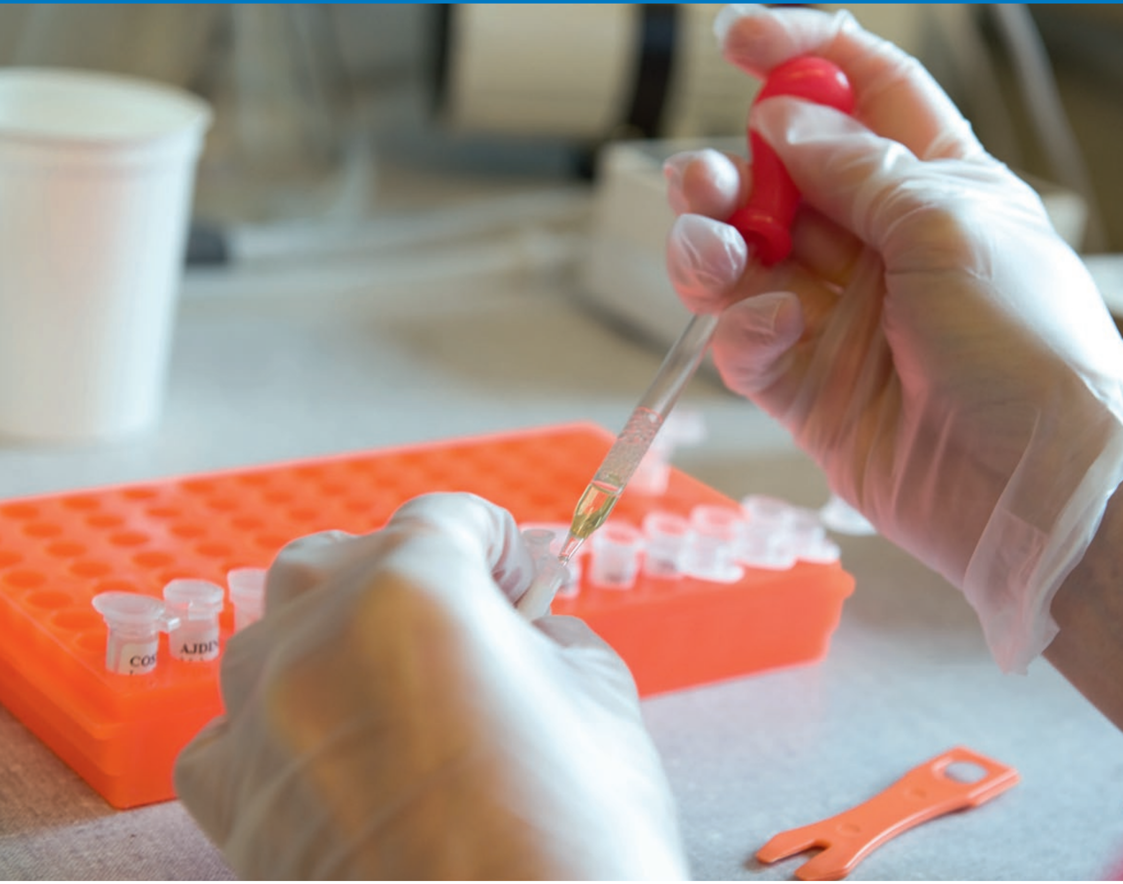

Hôpitaux Universitaires Genève

Geneva University Hospitals (HUG) and research

The HUG are proposing you assist bio-medical research by authorising the use of your medical data and biological samples. You are free to decide for yourself if you want to take part in this initiative or not. This brochure summarises the most important information that will assist you in your choice. Please take the time to read it. Do not hesitate to contact the people specified at the end of this brochure if you have any questions. You will then notify us of your decision in writing on the attached consent form. The consent that we request from you refers to your previous, current and future treatments at the HUG.

A University Hospital

Diagnosis and treatment of illnesses has progressed hugely over the last few decades. These breakthroughs are the result of many long years of bio-medical research in which doctors, scientists and patients have actively participated. As a University Hospital, the HUG also support high-quality research for the patients' current and future well-being. Thanks to this research, many people treated in our hospital have benefited from spectacular progress in the understanding and treatment of their illness. Despite this, there are still areas where knowledge on the causes, diagnosis and treatment of illnesses could be improved.

Precious medical information

When you receive medical treatment at HUG, we collect information related to your health, namely your age, gender, laboratory results, the illnesses that you suffer from, the treatments that you have been prescribed and sometimes specific genetic information. Similarly, biological samples – originating from tissues, blood and other body fluids – are often taken. This data and samples are used for diagnostic and/or treatment purposes.

Medical information is registered in your medical file. In contrast, in most cases, biological information is destroyed after the diagnosis and treatment of the illness. However, these materials, in particular when associated to your medical history, may prove to be very precious for research. Researchers may for example be able to use them to improve the diagnosis of an illness or to enable better tracking of responses to a treatment.

Large-scale participation is required

Research projects can only be developed if a high number of patients accept the use of their biological samples and health-related data for research purposes. Your assistance is therefore very important. It will enable researchers to acquire new medical knowledge and thus improve diagnosis and treatment options for a range of illnesses.

How can you **assist research?**

One day you may require treatment at the HUG to identify the cause of a health problem, undergo an examination or benefit from medical or surgical treatment. As a general rule, medical and care teams receive information on your present and past state of health and take biological samples if necessary. If you consent, this information and these samples can be made available to researchers.

What does “health-related data” mean?

This refers to all the data collected in your file for the purpose of treating your illness, such as information on possible risk factors and the results of clinical, radiological or laboratory examinations and genetic analyses. It also includes data describing the progress of the illness and the responses to any administered treatments.

What are my obligations if I consent to the research?

By giving your written consent you authorise researchers to use your health-related data and biological samples for research purposes. These are stored in a “biobank” located in the HUG. This biobank brings together all the data and samples of patients who have given their consent. Unless you revoke this consent, it shall continue to apply to all future research projects.

Furthermore, by giving your consent you accept that your data and samples will be transmitted to research institutes that work together with the HUG. You are not systematically informed when your biological materials and health-related data are used for research projects or transferred to another institution.

How to obtain information on your data and samples?

You have the right at any time to consult your medical data and request information regarding your biological samples which have been included in the HUG biobank. You can make this request in writing (see the practical information on the last page). You also have the right to be informed about current biomedical research projects at the HUG.

Protection of your data

Where are your data and samples stored if you give your consent for research?

We commit to store your medical data and samples securely. Specific regulations have been developed for the HUG biobank. These specify how your data and samples are coded and strictly govern access to all biological materials and data stored in the biobank.

How is your personal data protected?

Your data and samples are coded by the HUG before being included in the institutional biobank and made available to the researchers. Coding means that your surname, your first name and any information that could identify you are replaced by a code (in general a unique combination consisting of figures and letters). This means that people who do not know the code cannot connect the data to you.

The Federal Act on Research involving Human Beings (LRH) authorises research control and monitoring authorities (Cantonal Research Ethics Committee and Swissmedic, the Swiss Agency for Therapeutic Products) to access non-coded data, in other words, data that mentions the name of patients or enables their identification.

The representatives of these authorities are themselves subjected to professional secrecy.

What does data anonymisation mean?

Data that has been coded initially can be rendered anonymous by permanently and irreversibly deleting the code or the decoding key (the link between the unique code and the identity of the patient). After an anonymisation the link between the code and yourself can never be recovered. In other words, nobody will know that this data and samples are yours. At the HUG some data and samples included in the biobank are anonymised. In this case you may not request their destruction because they can no longer be identified as yours.

Use of your data

How is your data transmitted to the researchers?

All research projects conducted with samples and/or medical data must be pre-authorised by the Cantonal Ethics Committee. Samples and data may only be transmitted to researchers if they have been coded or anonymised. Research teams may be working in institutions such as hospitals, universities or private laboratories both in Switzerland and abroad. However, for data and samples to be sent abroad, the countries concerned must have legal provisions regarding data protection which are equivalent to those in Switzerland. The code enabling them to identify you is not sent to them in any case whatsoever.

What happens to the research results?

Your data and samples are studied in the research in "group" form along with those from other patients. Individual results are not analysed as they often have no, or only a slight, relevance for a particular patient. Therefore, giving your consent for research does not benefit you directly or personally. However, if a research project yields a result that proves to be very important for your health, you may be informed of this. However, these situations are very rare and patients are not generally informed individually of the research results. These results are generally published in medical reviews and may contribute to improving your own treatment and that of future patients.

What happens if you do not give your consent or you change your mind?

Your participation in this project is totally voluntary. If you withdraw your participation, this will not affect the care you receive at the HUG in any way at all. You may change your mind at any time even if you gave your consent initially. You do not need to justify your refusal to participate or your withdrawal. If you give your consent and then change your mind, the data and samples collected up to this time can no longer be used for research (except if they have been anonymised).

Practical
information

Your contacts

You may contact us at any time to obtain information on all your personal data. Should you have any questions or would like to find out more about this, please contact us at:

Centre de Recherche Clinique
Rue Gabrielle-Perret-Gentil 4
1211 Genève 14
☎ +41 (0)22 372 91 34
contact.crc@hcuge.ch

Réf. 458679 • Consentement général • Février 2017

This brochure has been compiled by a multi-disciplinary team in collaboration with the Head of Communications.

Declaration of consent for the re-use of health-related data and samples for HUG research purposes

After having acknowledged the information specified in this brochure and having received all the explanations you require, do you consent to the data from your medical file and your biological samples being used for research purposes?

Yes ☐ No ☐

- By signing you confirm that you have read the brochure with the detailed information and declare that you are sufficiently informed;
- You understand that your consent shall continue to apply for every new treatment at the HUG;
- You understand that you can withdraw your consent at any time without stating a reason;
- You understand that your materials and data are protected;
- You understand that your materials and data may only be transferred to third parties for research purposes if they are coded or anonymised;
- You understand that if, in exceptional circumstances, a research campaign revealed significant information for your health, you could be contacted again.

Surname, first name, date of birth or patient label

Location, date

Valid signature of the patient

Location, date

if required: signature of the legal representative (specify the relationship with the patient)

Should you have questions, please contact: Clinical Research Center
Rue Gabrielle-Perret-Gentil 4, 1211 Geneva 14
022 372 91 34, contact.crc@hcuge.ch

Hôpitaux Universitaires Genève

Février 2017 - anglais
